# Supplementary material for: Effectiveness and Safety of Ayurvedic Medicines in Type 2 Diabetes Mellitus Management: A Systematic Review and Meta-Analysis
Source: Front Pharmacol. 2022 Jun 8;13:821810. doi: 10.3389/fphar.2022.821810 (PMC9213670; doi:10.3389/fphar.2022.821810)
Supplement: Supplementary file 1 [file DataSheet1.zip › Supplementary Material/Forest and Funnel Plots/Zingiber officinale/TC.pdf]

Zingiber officinale - TC

Study	Intervention N	Intervention Mean	Intervention SD	Comparator N	Comparator Mean	Comparator SD	Mean Diff. with 95% CI	Weight (%)
Mahluji 2013	28	143.00	35.20	30	149.00	43.50	-6.00 [-26.45, 14.45]	14.24
Arablou 2014a, Arablou 2014b	33	173.10	28.70	30	194.00	45.10	-20.90 [-39.39, -2.41]	14.57
Azimi 2014, Azimi 2016	41	396.24	17.54	39	334.92	55.39	61.32 [43.50, 79.14]	14.68
Mozaffari-Khosravi 2014, Talaei 2017, Talaei 2018	40	186.84	33.52	41	179.81	32.69	7.03 [-7.39, 21.45]	15.20
Arzati 2017, Zarezadeh 2018	23	167.00	45.20	22	179.00	41.20	-12.00 [-37.30, 13.30]	13.35
Carvalho 2020	47	183.74	43.40	56	202.07	63.58	-18.33 [-39.77, 3.11]	14.06
Gholinezhad 2020	21	157.80	39.49	21	137.33	33.98	20.47 [-1.81, 42.75]	13.91
**Overall**							4.84 [-16.93, 26.61]	

Heterogeneity: $\tau^2 = 757.53$, $I^2 = 88.55\%$, $H^2 = 8.73$

Test of $\theta_i = \theta_j$: Q(6) = 54.91, p = 0.00

Test of $\theta = 0$: z = 0.44, p = 0.66

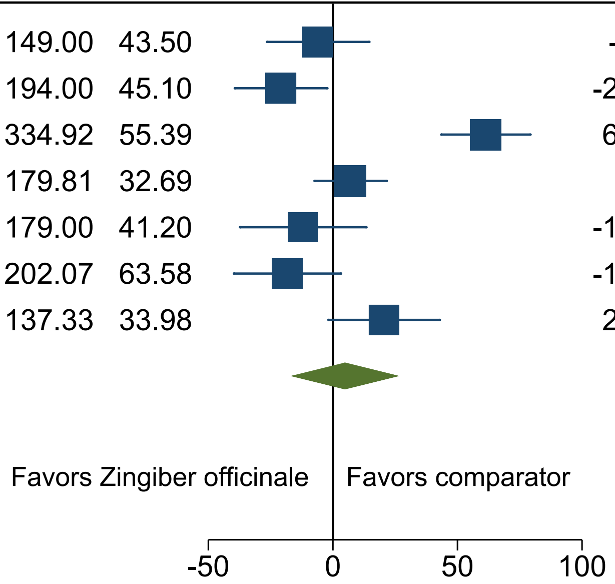

Random-effects REML model
